# Intraoperative radiotherapy electron boost in advanced and recurrent epithelial ovarian carcinoma: a retrospective study

**DOI:** 10.1186/1471-2407-11-439

**Published:** 2011-10-11

**Authors:** Ying Gao, Zi Liu, Xi Chen, Wei Luo, Long Zhang, Juan Wang

**Affiliations:** 1 The Department of Radiotherapy Oncology in the 1st Affiliated Hospital of Medical College of Xi'an Jiao Tong University, Xi'an, 710061, China

## Abstract

**Background:**

Relapses of epithelial ovarian carcinoma (EOC) have a poor prognosis and are almost always fatal. The aim of this study was to evaluate the clinical outcome and toxicity of intraoperative electron beam radiation therapy (IOERT) in advanced and recurrent EOC.

**Methods:**

Forty-five women with EOC were treated with IOERT. Twenty-five patients had primary disease (PD) without distant metastasis at IOERT, and 20 patients had an isolated local recurrence (ILR) after surgery. All 45 patients in this series underwent optimal cytoreductive (≤ 1 cm) surgery. The whole pelvic (WP) radiotherapy was intraoperatively delivered using 12 Mev electron beam; 43 patients received 18-20 Gy and two patients received 10 Gy. Thirty-three patients received postoperateive intraperitoneal (IP) chemotherapy, while seven patients received intravenous (IV) chemotherapy. Five patients refused concurrent chemotherapy. Overall survival (OS) rates were analyzed using the Kaplan-Meier method.

**Results:**

Tumor recurrence and metastasis were observed in 16 patients (35.6%). Of those, 14 patients (31.1%) relapsed and two patients (4.4%) had distant metastasis alone. Eight of 25 (32%) local failures were observed in the PD group, as compared to 6/20 (30%) in the ILR group (*P *= 0.885). Actuarial local control at five year follow-up was 31/45 (68.9%). Seventeen of the total 45 (37.8%) patients died. Nine of 25 (36%) in the PD group died, as compared to 8 of 20 (40%) in the ILR group. The 5-year OS and disease-free survival (DFS) rates were 28/45 (62.2%) and 25/45 (55.6%), respectively. In the PD group, the 5-year OS and DFS rates were 16/25 (64%) and 14/25 (56%) (*P *> 0.05, *vs*. the ILR group at 12/20 and 11/20, respectively). The OS and DFS in the IOERT plus IP group were 25/33 (75.8%) and 23/33 (69.7%), respectively, which were superior to the rates achieved with IOERT plus IV chemotherapy (*P *< 0.05, 2/7 and 1/7, respectively). The major complication of IOERT was neuropathy. Five (11.1%) patients developed peripheral neurotoxicity.

**Conclusions:**

IOERT may be feasible and effective as a boosting technique for advanced and recurrent ovarian cancer. IOERT plus IP chemotherapy may achieve high locoregional disease control and survival benefit with a low risk of toxicity. Peripheral nerves in the IOERT field are dose-limiting structures requiring nerve protection policies or a dose compromise to ensure against severe neurological damage.

## Background

Relapses of epithelial ovarian carcinoma (EOC) have a poor prognosis and are almost always fatal. The prognosis remains poor due to a high rate of recurrence. About 50-75% of women with ovarian cancer will develop persistent or recurrent disease [1]. The pelvis or abdomen is the initial recurrence site, accounting for approximately 85% of ovarian cancers [2]. Overall survival (OS) after recurrence depends on patient's performance status, and histological type and size of the relapse tumor [3-5]. Pelvis, peritoneum, liver, lung, lymph nodes, and central nervous system are the most frequent sites of relapse [6-8]. Platinum/paclitaxel-based chemotherapy is the current standard of treatment after surgical staging and resection of abdominal and pelvic cancers. Despite the advances in chemotherapy, however, the prognosis still remains poor since many patients develop abdominal or pelvic recurrence that is resistant to further chemotherapy. Radiotherapy has been shown to produce a response in chemo-resistant ovarian cancers, and may offer the possibility of improved tumor control.

Intraoperative electron beam radiation therapy (IOERT) is an innovative boosting technique used to deliver single high-doses of radiation (range of 10-20 Gy). One of the key advantages of this technique is the ability to irradiate selected anatomic areas that had been identified during the surgical procedure as high risk and/or residual disease sites, while avoiding surrounding dose-limiting structures. Thus, non-cancerous intra-abdominal organs can be protected from receiving full doses of irradiation, ultimately decreasing the incidence of severe enteritis and increasing the local control rates [9]. Studies of IOERT in multiple anatomical sites have produced valuable results in terms of locoregional control and toxicity [8,10].

It is generally anticipated that IOERT may represent an innovative treatment to improve the local control of EOC. Numerous non-randomized series trials over the past several years have demonstrated that IOERT was able to reduce the local recurrence rate of various cancers and had a positive impact on survival [11-13]. However, there is a paucity of literature discussing the utility of IOERT as a modality for treatment of ovarian carcinoma. The aim of this study was to retrospectively review all cases of EOC that were treated with IOERT at our center over the past ten years in order to evaluate the clinical efficacy of IOERT. We specifically addressed patient outcome and IOERT toxicity, so as to suggest the best parameters for its use as a therapeutic strategy.

## Methods

### Patients

This study was a non-randomized trial and included retrospective analysis of 45 women with EOC who were treated with IOERT at the 1st Affiliated Hospital of the Medical College of Xi'an Jiaotong University between January 2000 and January 2010. Twenty-five patients had primary disease (PD) without distant metastasis at IOERT, and 20 patients had an isolated local recurrence (ILR) after surgery. Patients with prior postoperative adjuvant treatments, such as chemotherapy, were included in the analysis. The present study was approved by the ethics committee of the 1st Affiliated Hospital of Medical College of Xi'an Jiaotong University. All patients had provided written informed consent for the IOERT procedure. Inclusion criteria were a history of EOC, diagnosed by clinical examination and CT scan, with histological evidence. The following features were studied: initial stage by standards of the International Federation of Gynecology and Obstetrics (FIGO), initial performance status, CA125 level, response to IOERT and chemotherapy, time and sites of relapse, OS and disease-free survival (DFS). Disease progression was defined as: new lesions, consistent with new sites of disease, detected by imaging, including CT, magnetic resonance imaging (MRI), ultrasound and/or plain X-ray; new elevation of CA-125; biopsy/histology of new lesions and new signs upon clinical examination, or symptoms consistent with new sites of disease (Table 1).

**Table 1:** Patient Characteristics

	Total	PD	ILR
Cases	45	25	20
Histology type			
serous adenocarcinoma	36	21	16
papillary adenocarcinoma	9	4	4
CA-125 level			
≥ 35 U/ml	38	20	18
< 35 U/ml	4	3	1
unkown	3	2	1

### IOERT procedure

All 45 patients in this series underwent optimal cytoreductive (≤ 1 cm) surgery. The whole pelvic (WP) radiotherapy was delivered intraoperatively using 12 Mev electron beam (Varian 1800). Forty-three patients received 18-20 Gy and two patients received 10 Gy. The superior border of the field was at the bifurcation of common iliac vessels, while the inferior border covered 2 cm inferior to the operated vaginal vault, and laterally 1 cm beyond the lateral margin of external and common iliac vessels. The electron intraoperative applicator was 10-12 cm in diameter. The bladder, intestines, and sigmoid colon were shifted out of the radiation field, and the rectum was shielded with a 6 mm thick lead sheet. The portion of the obturator nerve in the pelvic region was partially shifted out of the radiation field, as allowable.

### Postoperative therapy

Our institution had implemented a single day outpatient intraperitoneal (IP) chemotherapy regimen following IOERT, based upon careful review of the most current published data. Every three weeks 100 mg/m^2 ^cisplatin was administered, for an intended six cycles. Thirty-two IOERT patients received four to six courses of the IP chemotherapy regimen. Seven patients received intravenous (IV) chemotherapy using 80 mg/m^2 ^cisplatin, 50 mg/m^2 ^adriamycin, and 500 mg/m^2 ^cyclophosphamide; the IV regimen was designed to treat patients once every 21 days for an intended six cycles. One patient was treated with two courses of IP chemotherapy plus IV chemotherapy. Five patients refused chemotherapy altogether (Table 2).

**Table 2:** Therapy Types

Therapy Types	Total	PD	ILR
IOERT+IP(4-6 courses)	32	17	15
IOERT+IP(2 courses)	1	0	1
IOERT+IV	7	5	2
IOERT	5	3	2

### Evaluation of acute and late toxicities

Acute and late toxicities were graded by the Common Terminology Criteria for Adverse Events (CTCAE v3.0) and the Radiation Therapy Oncology Group/European Organization for Research and Treatment of Cancer (RTOG/EORTC) criteria, respectively. Complications that occurred within 90 days of the start of primary treatment were considered to be acute complications, and those that occurred more than 90 days after the start of treatment were considered to be late complications.

### Statistical analysis

Overall survival and disease-free survival curves were calculated according to the Kaplan-Meier method. A *P*-value of < 0.05 was considered significant for all statistical analysis.

## Results

### Patient characteristics

The median age of the 45 patients at the time of EOC diagnosis was 52.9 years (range: 42-62 years). Twenty-five patients were treated for PD and 20 patients for ILR. All patients of the primary tumor group were positive for peritoneal cytology and were diagnosed as FIGO stage III. CA-125 serum levels were elevated in 38 patients, normal in four patients, and non-assessed in three patients. Histology of the adenocarcinoma was serous in 80% (36 patients) and papillary in the remaining 20% (9 patients) (Table 1). Abnormal CA-125 (defined as a level > 35 U/mL) was observed in 84.4% of patients at pre-treatment, 64.4% after IOERT and one cycle of IP chemotherapy, 15.6% after three cycles of IP chemotherapy, and 6.7% at the end of chemotherapy.

During the follow-up interval, 17 (37.8%) patients died. Thirteen (28.9%) died of tumor-related disease, and four died of causes other than cancer. Tumor recurrence and metastasis were observed in 16 patients (35.6%). In the entire group, 14 patients (31.1%) relapsed and two patients (4.4%) had distant metastasis alone; of those two, one had abdominal relapse and lung metastasis, and the other had lung, liver, and spleen metastases. Among them, isolated local recurrence was detected in 11 patients (24.4%), and combined local failure and distant metastasis was detected in three patients (6.7%). Eight of the 25 (32%) local failures were observed in the PD group, compared to those in the ILR group (6/20, 30%; *P *= 0.885). Actuarial local control at five years follow-up was 31/45 (68.9%). The rates of distant metastasis were similar among the two groups (3/25 in PD and 2/20 in ILR; *P *= 0.832). The mean times to recurrence in the PD and ILR groups were eight months (range: 7-11 months) and 10 months (range: 3-34 months), respectively. Ten of the 14 local failures were determined to be locoregional failure in the abdomen tissue, outside of the IOERT targeted region.

### OS and DFS

**Figure 1:**
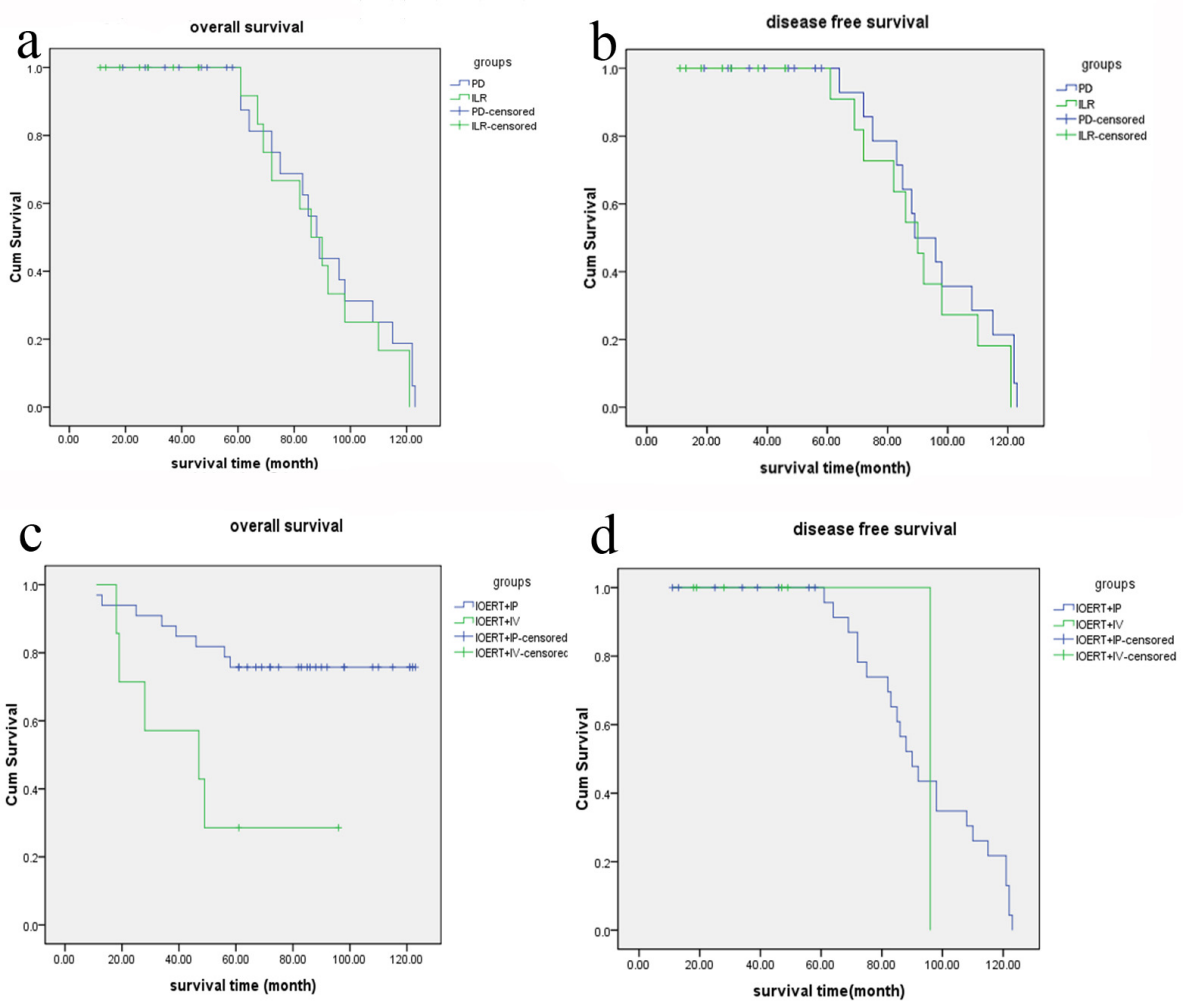
**Survival analysis in advanced and recurrent epithelial ovarian carcinoma after IOERT**. a Overall-survival curve stratified for PD versus ILR (P > 0.05). b Disease-free survival survival curve stratified for PD versus ILR (P > 0.05). c Overall-survival curve stratified for IOERT+IP versus IOERT+IV (P < 0.05). d Disease-free survival curve stratified for IOERT+IP versus decreased IOERT+IV (P < 0.05).

The mean follow-up time was 78 months (range: 11-123 months). During that time, 17 of 45 (37.8%) patients died of disease. Nine of the 25 patients (36%) in the PD group died, as compared to eight of the 20 patients (40%) in the ILR group. The 5-year overall survival and disease-free survival rates were 28/45 (62.2%) and 25/45 (55.6%), respectively. In the PD group, the 5-year OS and DFS rates were 16/25 and 14/25 (*P *> 0.05 *vs*. the ILR group at 12/20 and 11/20, respectively) (Figure 1a and 1b). The OS and DFS in the IOERT plus IP group were 25/33 and 23/33, which were superior to those in the IOERT plus IV group (2/7 and 1/7, respectively; *P *< 0.05) (Figure 1c and 1d).

### Toxicities

Generally, IOERT was well tolerated. The toxicities noted included only those potentially related to local effects from local treatments. Toxicity related to chemotherapy was not noted. No radiation-induced nausea or vomiting was observed. No delayed wound healing occurred in any of the patients. No patients with abdominal relapse suffered small bowel obstruction during the entire follow-up period.

The major complication was neuropathy. Five (11.1%) patients developed neurotoxicity; grade1 and 2 peripheral neuropathy was cited most frequently. The median time to the onset of neurotoxicity was 14 months (range: 8-22 months). In three cases, the nerve had been included in the IOERT field, indicating the chance of developing neuropathy was 16.7% if the peripheral nerve had been included in the IOERT field. In two out of 27 patients (7.4%), the nerve had not been irradiated. In addition, one patient suffered abdominal pain (2.2%) and two patients (4.4%) developed hydronephrosis.

Other toxicities, such as urinary tract infection (2.2%), intestinal injury (4.4%) and crura edema (2.2%) were reported as related to surgery. All cases were sufficiently managed by conservative treatment.

## Discussion

Ovarian carcinoma is the second most common gynecologic cancer and the leading cause of death from gynecologic malignancy. Epithelial ovarian cancer represents the primary cause of death from gynecological cancer in Western countries, with approximately 26000 new cases diagnosed in the United States each year [14-17]. More than two-thirds of patients with epithelial ovarian cancer are diagnosed in an advanced stage of disease at presentation because of the absence of specific signs and symptoms. The 5-year overall survival ranges from 89% for stage IA to 13% for stage IV disease, according to the annual report from FIGO [18]. About 40-85% of patients who have stage II to IV disease will relapse after primary therapy and develop abdominal or pelvic recurrence; these tumors are also characterized by a low response rate to further chemotherapy and subsequent poor prognosis (5-year survival rate < 25%) [19,20]. Invasion of the ovarian capsule and dissemination in the peritoneal cavity is the main route by which ovarian carcinoma spreads [21], accounting for about 82% of cases; in contrast, only 12% of ovarian metastatic events involve the retroperitoneal lymph nodes [19].

Platinum/paclitaxel-based chemotherapy is the current standard of treatment after surgical staging and resection of abdominal and pelvic cancers. Despite the significant advances in treatment, however, the prognosis remains poor since the standard therapy does not lead to a sufficient reduction of tumor cells and fails to cure. Abdominal radiotherapy offers the possibility of improved tumor control; moreover, the potential role of radiotherapy for improving disease control in the abdomen and pelvis may increase the disease-free interval and survival [22]. To date, however, there has been no proven benefit and there is significant toxicity associated with this treatment. Thus, an alternative effective therapy is urgently needed.

IOERT improves the therapeutic ratio by decreasing the toxicity in dose-limiting normal tissues that can be displaced or protected. It can be administered as an upfront radiation boost, simultaneously with surgical resection, which might allow total electron beam radiation therapy (EBRT) dose to be decreased without jeopardizing local control or survival. Another possible advantage of IOERT is that it might indirectly improve the quality of therapy by decreasing the overall treatment time as a secondary endpoint [23]. IOERT clinical trials have also been mainly conducted on patients with locally advanced malignancies in the abdomen and pelvis [8,24]. In this study, we observed that it was especially effective in patients who had an adequate resection of their localized extraperitoneal recurrence and a significant survival benefit whether the patients had primary disease or not. The 5-year overall survival rates were 64% in the PD group and 60% in the ILR group. Non-IORT approaches have been reported in the literature as producing OS rates of 20.6% for primary ovarian patients (stage III) and < 25% for recurrent cases [19,20,25].

Among the 14 patients with local failures in our study, a significant proportion (10/14) of the relapse sites were found outside of the IOERT targeted region. In addition, with the low toxicity associated with IOERT, the quality of life was considered an important endpoint in these patients, which is particularly dependent on strategies providing high local control rates and organ preservation, such as bladder, intestines, sigmoid colon, and the pelvic portion of the obturator nerve). Moreover, in this series we have shown that disease limited to the local and regional areas can be successfully treated with significant overall survival and disease-free survival. Even though ovarian cancer is known to have high incidence of widespread metastases, this is not always the case.

Our regimen also exhibited a favorable survival time, even in cases of abnormal CA-125 levels at pre-treatment and obvious decline in CA-125 serum level following tumor resection, IOERT, and IP chemotherapy. A study by Krivak *et al*. found that patients with an abnormal CA-125 (> 35 U/mL) prior to treatment were 2.45 times more likely to experience disease progression and 2.78 times more likely to die of disease, as compared to patients with CA-125 < 35 U/mL [26].

Although the absolute number of toxicities was lower in the present study, the incidence rate of neurotoxicity seems high. Neuropathy is a dose-limiting toxicity in IOERT and other anatomic sites treated with this modality. Animal studies have shown that the tolerance of nerve structures to IOERT may be lower than 15 Gy [15]. In the present series, 11% of the patients developed symptoms associated with peripheral neuropathy. In four of these patients, substantial improvement was observed over time, with successful recovery noted at prolonged follow-up. Peripheral nerves in the IOERT field are dose-limiting structures requiring a dose compromise or the activation of the nerve protection policies in the IOERT component to avoid severe neurological damage.

In addition, the findings from this study suggested that subsequent IP chemotherapy may have improved the local control rates. This is consistent with a report by Chin *et al*. [27]. Combined treatment modalities increase the effect of radiation significantly. IP chemotherapy was first proposed in the 1970s as a way to maximize drug delivery to the tumor while avoiding systemic toxicities associated with IV administration of the same agents [28-30]. The results of the Gynecologic Oncology Group (GOG-172) phase III trial demonstrated that bidirectional chemotherapy using IV paclitaxel plus IP cisplatin and paclitaxel significantly improved survival in patients with optimally debulked stage III disease [1]. Based on these results, the National Cancer Institute and GOG have issued a clinical announcement recommending that patients with stage III ovarian cancer should be considered for IP chemotherapy after undergoing optimal surgical cytoreduction [31]. Owing to the unique properties of the peritoneum, IP chemotherapy affords the opportunity to use higher concentrations of drugs for prolonged periods of time, directly bathing resected tumor beds, lymph node basins, and residual tumor nodules in the therapeutic agent. Unfortunately, IP chemotherapy is still limited by the fact that it cannot penetrate into large tumor nodules, essentially 3 mm or greater; IOERT may be able to do this, however [32]. Our retrospective study suggests that IOERT plus IP chemotherapy may be a useful treatment for selected patients with EOC. Therefore, with the encouraging results of this report, IOERT plus IP chemotherapy should be further studied for its utility in chemoresistant patients with recurrent ovarian cancer.

Although IOERT has been recommended for recurrent cervical cancer by the National Cancer Institute [33], to our knowledge, the present report represents the first systematic review of the activity of IOERT plus IP chemotherapy against EOC. The limitations of this report are those associated with any retrospective study, including potential referral bias, other types of selection bias, and a variety of treatments, doses, and schedules. In addition, IOERT in ovarian carcinoma has not been investigated in detail, and this research did not reach statistical significance because of the small numbers in this series; nonetheless, we did observe a marked advantage in overall survival and disease-free survival. We have also demonstrated that the involved field of radiation therapy was relatively unaffected in this heavily pretreated population.

The findings of this study confirm our clinical impressions and provide important information with which to move forward in developing better therapies for advanced and recurrent carcinoma of the ovary. Additionally, the current systemic therapy options are all associated with toxicities that are potentially detrimental to a patient's overall quality of life or well-being. The IOERT treatment provided good prognosis of EOC, and in some cases with postoperative IP chemotherapy. A prospective randomized control trial comparing IOERT with various chemotherapies, established or candidates for EOC, should be considered. Our findings also suggest the need for larger studies to determine the role of IOERT in local and regional control and to evaluate its impact on distant metastasis and overall survival in advanced and recurrent ovarian cancers.

## Conclusions

IOERT may be feasible and effective as a boosting technique to treat advanced and recurrent ovarian cancers. IOERT plus IP chemotherapy may achieve high locoregional disease control and survival benefit with a low risk of toxicity. However, careful attention should be paid to peripheral nerves as specific IOERT dose-limiting structures.

## Competing interests

The authors declare that they have no competing interests.

## Authors' contributions

YG was the guarantor of integrity of the entire study and drafted the manuscript. ZL designed and participated in the research, data analysis and manuscript editing. XC participated in the design of the study and performed the statistical analysis. WL carried out the studies and participated in manuscript preparation. LZ and JW participated in data analysis. All authors read and approved the final manuscript.

## Pre-publication history

The pre-publication history for this paper can be accessed here:

http://www.biomedcentral.com/1471-2407/11/439/prepub
